# Central nervous system involvement in mycosis fungoides: relevance of tcr gene testing in cerebrospinal fluid

**DOI:** 10.1186/2193-1801-3-29

**Published:** 2014-01-17

**Authors:** Elisa Giorli, Elisabetta Traverso, Luana Benedetti, Simona Zupo, Bruno Del Sette, Giannamaria Cerruti, Massimiliano Godani

**Affiliations:** Department of Clinical and Experimental Medicine, Pisa University, Medical School, Pisa, Italy; Neurology Unit, St’ Andrea Hospital, La Spezia, Italy; Clinical Chemistry and Microbiology, Sant’Andrea Hospital, La Spezia, Italy; Molecular Diagnostic Unit, IRCCS AOU San Martino-Institute of National Cancer Research, Genova, Italy; Department of Neuroscience, Rehabilitation, Ophthalmology, Genetics, Maternal and Child Health, University of Genoa, Genoa, Italy

**Keywords:** Mycosis fungoides, CNS involvement, PCR, Cerebrospinal fluid, CSF, PCR analisys

## Abstract

**Introduction:**

Mycosis Fungoides (MF) is a rare malignant T-cell lymphoma, involving mainly the skin. In 50%–75% of cases, it can involve organs other than skin, with a 11%–14% Central Nervous System involvement (CNS).

**Case report:**

A 82-year-old woman presented to our Department with a 15-years history of MF with skin lesions. Neurological examination showed dysarthria and a left facio-brachial-crural hemiparesis. A CT scan showed a right fronto-rolandic lesion. A MRI, including DWI, confirmed the presence of the “neoplastic” lesion with slight hemorrhagic component and leptomeningeal contrast enhancement. Molecular TCR rearrangement test by PCR analysis was performed on skin biopsy, showed the presence of a single peak which fits with a monoclonal TCRG gene rearrangement (size 67). Molecular TCR test was also performed on the cerebrospinal fluid (CSF), which confirmed the presence of lymphocyte clone T g/ more expressed with the same size of that observed in the skin biopsy A total body CT scan did not show any lymphnodal or extranodal disease. The patient died after ten days.

**Conclusion:**

MF usually occurs in the context of advanced and often histologically transformed cutaneous disease. Isolated CNS involvement is remarkably rare. This case highlights the need for regular neurologic follow-up after the diagnosis of MF, in particular when features that suggest risk of disease progression are present. Furthermore, the analysis of the skin biopsy and above all of CSF by PCR technique, based on our experience, should always be executed in MF patients with signs or symptoms suggesting CNS involvement.

Mycosis Fungoides (MF) is a rare malignant T-cell lymphoma which mainly involves the skin. Generally, these are indolent tumors, with a median survival rate of 8 to 9 years and they occur more commonly in men older than 50 years. Autopsy studies have demonstrated that the disease may evolve into a generalized lymphoma involving lymph nodes, lung, heart, spleen, and gastrointestinal tract in approximately 50 to 75% of patients who have died of MF and central nervous system (CNS) involvement in 11 to 14%. Nevertheless, an autopsy study reported central nervous system (CNS) *involvement* in approximately 11 to 14% of patients died for MF. (Zonenshayn et al; 1998).

We report a case of a 82 years old woman with a right fronto-rolandic lesion due to MF localization, in which the diagnosis was done mainly with PCR analysis of gene rearrangements in the cerebrospinal fluid.

A 82-year-old woman was admitted to our Department for generalized seizure and left-sided sensory-motor deficit. Personal history reported a 15-years lasting MF, with isolated skin involvement. She reported paresthesia and motor weakness of her left arm starting 10 days prior to her hospitalization and arrived the Emergency Room after a generalized tonic-clonic seizure. Physical examination showed two large infiltrated and ulcerated skin lesions on her right leg (Figure 1). Neurological examination showed mild dysarthria and left sensory-motor deficit. Cerebral CT scan showed a right fronto-rolandic hypodense lesion. Brain MRI, including DWI, confirmed the presence of a proliferative lesion with a slight hemorrhagic component and leptomeningeal contrast enhancement (Figure 2). An EEG showed polymorphic theta waves in the right temporal region. The skin biopsy showed dermal infiltrate, primarily containing lymphoid T-cells with cytological atypia and immunophenotype CD3+, CD45+, focally CD 56-/+, CD30-, ALK-, EBV-, myeloperoxidase-, TDT-, CD4-, CD8-. Relying on previous studies (Lally et al. 2007), a molecular TCR rearrangement test with PCR analysis was performed on the skin biopsy, that showed the presence of a single peak which fits with a monoclonal TCRG gene rearrangement (size 67).

**Figure 1:**
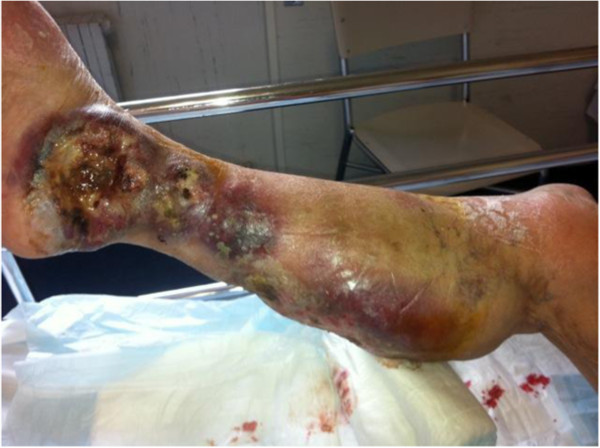
**Skin lesion on the right leg of patient.**

**Figure 2:**
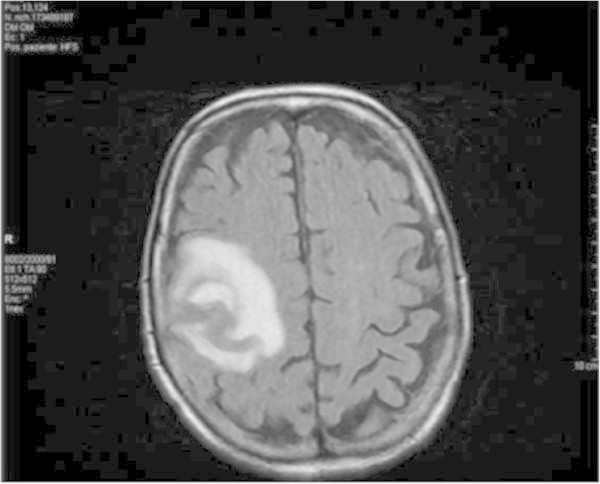
**MR imaging showing a proliferative lesion on right fronto-rolandic region.**

A molecular TCR test was also performed on the cerebrospinal fluid (CSF) using different primer such described in previous studies (Van Dongen et al; 2003), and confirmed the of expression of clone T g/ of the same size of the ones observed in the skin biopsy (Figure 3). A total-body CT scan did not show any other lymphnodal or extranodal disease. The clinical course was very severe and the patient died after ten days.

**Figure 3:**
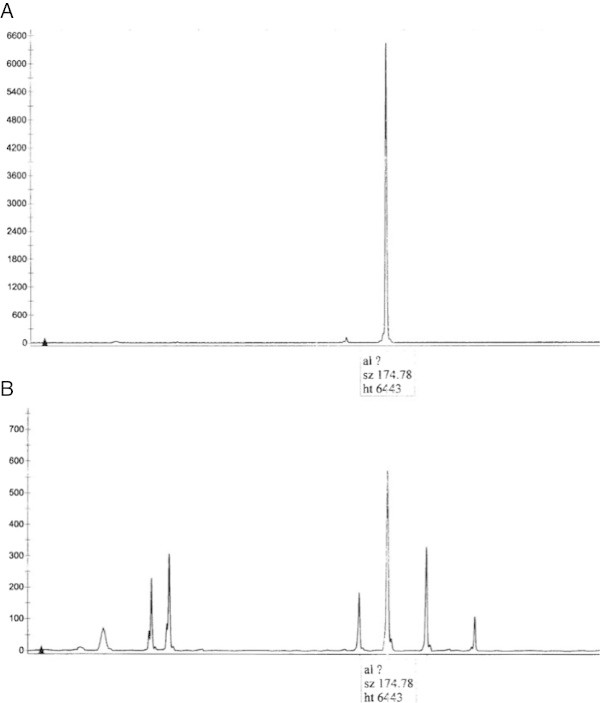
**T cell clonality testing by PCR analysis of TCR Gamma gene rearrangements.** TCR clonality profiles were obtained by extracted DNA from the diagnostic tissue for MF **(A)** and from cells **(B)** derived from CSF. The arrows indicate the TCRG monoclonal rearrangement.

CNS involvement in MF usually occurs in the context of advanced and often histologically transformed cutaneous diseases. At presentation, the disease is usually limited to the skin, with lesions that resemble eczema or psoriasis. Later on it can spread to the deeper layers of the derm, with the possibility of lymph nodes involvement; finally, visceral involvement occurs, yet often subclinical (Bruggermann et al; 2007). Lymph nodes are primarily involved in 75% of cases, followed by lungs (66%), liver (53%) and spleen (60%), although often multiple organs are affected (Weinstock and Reynes 1999).

There are few studies dealing with risk assessment and clinical course in patients with neurological symptoms due to MF. One study reported that nine patients out of 680 consecutive newly diagnosed cases of MF (1.3%) were found to have neurological involvement during follow-up. All of them showed severe courses of neurological disease (Weinstock and Reynes 1999). CNS involvement is observed within an average of 3–5 years from the initial diagnosis, typically occurring in patients with advanced infiltration of other organs (Stein et al. 2006), with autopsy demonstrating CNS involvement in 11–14% of cases (Zonenshayn et al; 1998). During life, CNS involvement is unusual, having been observed in 1.6% of 187 patients with cutaneous T-cell Lymphoma in one single series (Guilloton et al. 2001).

In 2 autopsy series consisting of 131 patients who died as a result of MF, the most common form of CNS involvement entailed the meninges (Kaufman et al. 1994), and only nine patients (6.8%) had involvement of the brain parenchyma. In approximately half of the patients with CNS MF a single area is affected, usually close to meninges; in the other half of patients it is more likely to have a “cerebral invasion” (Epstein et al. 1972).

The most common symptoms of CNS involvement in MF are confusion, nausea, headaches, gait difficulties, lethargy and weakness (Greene 1979), (Bodensteiner and Skikne 1982). In conclusion, isolated CNS involvement in MF without evidence of systemic disease is remarkably rare and only few cases are described in literature. Moreover, this is the first case in which diagnosis of certainty was made with the combined PCR analysis of CSF and skin biopsy.

This case highlights the need for regular neurologic follow-up after the diagnosis of MF, particularly in those patients that are showing a progression of the disease. We finally suggest to perform PCR analysis of skin biopsy together with CSF in patients with CNS lesions and Mycosis Fungoides.

## Informed consent

Written informed consent was obtained from the patient for the publication of this report and any accompanying images.

## References

[CR1] Bodensteiner DC, Skikne B (1982). Central nervous system involvement in mycosis fungoides. Cancer.

[CR2] Bruggermann M (2007). Powerful strategy for polymerase chain reaction based clonality assessment in T cell malignancies. Leukemia.

[CR3] Epstein E, Levin DL, Croft JD, Lutzner MA (1972). Mycosis fungoides: survival prognostic features, response to therapy, and autopsy findings. Medicine (Baltimore).

[CR4] Greene MH (1979). Mycosis fungoides: epidemiologic observations. Cancer Treat Rep.

[CR5] Guilloton L, Drouet A, Estival JL (2001). Transformation of mycosis fungoides to pleomorphic T-cell lymphoma and central nervous system involvement. Rev Med Int.

[CR6] Kaufman DC, Habermann TM, Kurtin PJ, O’ Neill BP (1994). Neurological complications of peripheral and cutaneous T-cell lymphomas. Ann Neurol.

[CR7] Lally A, Hollowood K, Whittaker S, Turner R (2007). Central nervous system involvement in stage 1b mycosis fungoides. Br J Dermatol.

[CR8] Stein M, Farrar N, Jones GW (2006). Central neurologic involvement in mycosis fungoides: ten cases, actuarial risk assessment, and predictive factors. Cancer J.

[CR9] Van Dongen JJ, Langerak AW, Brüggemann M, Evans PA, Hummel M, Lavender FL, Delabesse E, Davi F, Schuuring E, García-Sanz R, van Krieken JH, Droese J, González D, Bastard C, White HE, Spaargaren M, González M, Parreira A, Smith JL, Morgan GJ, Kneba M, Macintyre EA (2003). Design and standardization of PCR primers and protocols for detection of clonal immunoglobulin and T-cell receptor gene recombinations in suspect lymphoproliferations: report of the BIOMED-2 Concerted Action BMH4-CT98-3936. Leukemia.

[CR10] Weinstock MA, Reynes JF (1999). The changing survival of patients with mycosis fungoides: a population-based assessment of trends in the United States. Cancer.

[CR11] Zonenshayn M, Sharma S, Hymes K, Knopp EA, Golfinos JG, Zagzag D (1998). Mycosisi fungoides metastasizing to the brain parenchyma: case report. Neurosurgery.

